# Medical Items

**Published:** 1896-05

**Authors:** 


### *MEDICAL ITEMS.*

---

How dear to our heart is the cash on subscription,
When the generous subscriber presents it to view;
But the man who don't pay—we refrain from description,
For perhaps, gentle reader, that man may be you.—*Exchange.*

---

THE Regular Board of Examiners rejected seven applicants for license in April.

---

DRS. K. L. REID, and E. C. Cartledge have been appointed internes upon the staff of the Grady Hospital.

---

THE art of surgery will never advance till professional men have courage to publish their failures as well as their successes.—*John Hunter.*

---

DR. THEODORE LAMB, of Augusta, Professor of the Principles of Medicine in the Medical College of Georgia, died April 15th, in the thirty-ninth year of his age.

---

A WRITER says, in an exchange, "For urticaria moisten the wheels with cold water, and rub with salt," etc. The name of the vehicle thus afflicted with urticaria is not mentioned.

---

IT is proposed to hold the next meeting of the American Medical Association in Philadelphia. The first meeting of the Association was held in Philadelphia forty-nine years ago, and it seems eminently proper that the semi-centennial of the Association should be celebrated in the same place.

THE *National Formulary,* supplement to the *National Dispensatory,* is kindly sent us by the publishers, Lea Brothers & Co., Philadelphia. The *Formulary* contains a complete list of unofficial preparations, with full directions for making the same, and should be owned and used by all who have to prepare and dispense medicines.

---

WE take pleasure in announcing a series of valuable articles by Dr. Thos. F. Rumbold, of St. Louis, on the Pathology of the Mucous Membrane of the Nose and Throat in Infants, Children, and Adults. The first paper appears in this issue, and three or four others will be published in succeeding issues. We think our readers will find them interesting and instructive.

---

BROTHER DANIELS, of the Texas *Medical Journal,* is having some vexation of spirit over his printer. By the way, we wonder if this same printer is not responsible for the amusing allusion to that charming book, "*Under* the Bonny Brier Bush," and for the suggestion that "brier" should be spelled with an *a*. Surely Brother Daniels knew better than this. Touch up your printer again, Brother Daniels.

---

ON March 24th, Senator Gallinger, of New Hampshire, presented petitions and papers from medical men, clergymen, and educators to the United States Senate in favor of setting apart a Government reservation for the benefit of persons in the North suffering from pulmonary diseases, who might be benefited by a change of climate to the salubrious atmosphere of the Rocky-Mountain region. He subsequently introduced a bill setting apart the Fort Stanton Military Reservation in New Mexico.

---

FRANK LESLIE'S POPULAR MONTHLY is now running an interesting series on the "Lees of Virginia." The introductory article was written by Mrs. Roger A. Pryor upon "The Ancestors of General Robert E. Lee," in England and in Colonial Virginia, and the times in which they lived. This article, and those to follow, will be profusely illustrated, the whole forming an authoritative pictorial chronicle of the distinguished family which has given to America so many statesmen, warriors, and types of chivalrous patriotism.

---

BEGINNING with April, the Virginia *Medical Monthly* becomes a *Semi-Monthly*. The monthly was organized more than twenty years ago, and under the constant and excellent management of Dr. Edwards, it has always occupied a foremost place in our journalism. The change to a semi-monthly is a change in the right direction, which we hope will still further increase our contemporary's usefulness and prosperity. The first issue of the *Semi-Monthly* (some bad proof-reading excepted) gives promise of being a distinct improvement on the *Monthly*. Success to the *Semi-Monthly*.

---

ACCORDING to the *Medical Record*, the following kinds of medical men are to be avoided, and we will add, not to be imitated: "The one who has acute exacerbations of insanity when exposed to any one new fad. The one who always sees hundreds of cases of a rare disease. The one who can always match your case and improve on your treatment. The one who always finds you have omitted something in the examination of your case. The one who thinks he can talk well, and is always ready to discuss any paper of the evening. The one who is always ready to do the new operation. The one who is in a chronic fear of being anticipated in his important discoveries. The one who in consultation feels it his conscientious duty to explain to the patient why he differs with the attending physician."

---

IN St. Louis, after the famous trial and conviction of Duestrow, lately, an expert for the defense said: "In view of the preponderance of evidence going to establish the insanity of the defendant, the verdict was really a great surprise."

An expert for the State said: "In view of the preponderance of evidence going to establish the sanity of the defendant, the verdict was only what was expected."

The jury said: "The evidence given by the medical experts on either side of the case had no bearing on the jury in rendering a verdict."

Such is fame! But the expert testimony in this case is said to have cost nearly $20,000.

---

Professor Billson says that in well defined secondary syphilis some form of mercury will be the best treatment.

Professor Air says that in renal colic there is apt to be pain over the affected spot, or thereabouts. A hypodermic of morphine will probably give relief. He also says that every pain in the side is not appendicitis.

Professor Air says that a useful and efficient laxative is sulphate of magnesium—a quarter of a pound for breakfast.

Professor Burst says that the new coal-tar product, amidopyrophosphatemethylaminehydroxide, sixty grains every two hours, is the best remedy for subinvolution of the umbilicus after labor.

Professor Steen says that one should not do a laparotomy within two hours after performing a post-mortem or dissecting a cadaver. After a laparotomy, the operator should see that all sponges, forceps, needles, scissors, eye-glasses, lead-pencils, etc., are removed from the abdominal cavity. One cannot be too careful about such things.—"*Class-Room Notes*" *from Bunglesome's Clinical Recorder.*

---

The New York *Polyclinic* has lately "touched lightly and compassionately upon the self-imposed ignorance of certain of our brothers in medicine," and has thoroughly proven the existence of such ignorance by certain quotations from medical journals. Thus, one writer describes his treatment of malignant diphtheria: "Fumigation with alcoholic tincture of eucalyptus glob. in boiling water. Restored the action of the skin with aconite, antidoted the poison already generated with echinacea and eucalyptus. Protected the kidneys with equisetum. Supported the action of the heart with tincture of the flowers of phyllo cactus blossoms." In a case of "well mitigated" scarlatina anginosa, the same treatment was pursued and the "soda bath, aconite and cactus, with attention to gargle, kept it mostly to the surface." Another contributor described an awful case of lead-poisoning caused by the chewing of a *lead-pencil.* The *Polyclinic* has done right in calling attention to some of these ridiculous pieces sometimes seen in journals, and it is equally right in the conclusion based upon them that practitioners need to be continually renewing their education. We can duplicate from some of our own rejected manuscripts equally absurd ideas.

---

In November, 1895, the Parish Medical Society of New Orleans, La., appointed a commission for the investigation and a public test of Antiphthisin as to its value in tuberculosis, to be made in the Charity Hospital of New Orleans, the commission consisting of the following members: Dr. Edmond Souchon, Dr. A. J. Bloch, Dr. J. D. Bloom, Prof. John B. Elliott, Professor R. Matas, Professor F. W. Parham, Dr. F. Loeber, Dr. Chas. Chassaignac, Dr. John H. Bemis, Dr. Joseph Holt, Dr. H. L. Lewis, Dr. P. E. Archinard, Dr. O. L. Pothier, Dr. A. McShane, Dr. C. J. Landfried.

The treatment of cases began November 27th, and its final report was presented to the Parish Medical Society at its regular meeting, March 28, 1896. The following are the conclusions arrived at:

*In Surgical Cases.*—A consideration of the three improved cases would certainly lead us to believe that Antiphthisin has decided value, and we should commend its careful, tentative employment in such cases in conjunction with general measures, and the usual, appropriate surgical operative treatment. The glandular case we consider especially encouraging. This case would seem to have required a most serious operation for the removal of the glands, with great uncertainty of ultimate benefit. The improvement under Antiphthisin treatment would alone justify us in ascertaining that we have in this remedy a most valuable aid in the management of such cases. The hypodermic employment of the remedy would seem to be especially advantageous, if administered under careful aseptic precautions.

*In Medical Cases.*—1. In nearly every case the area of lung involved decreased, if it did not clear up entirely.

2. Ascultation bore out the results of percussion, vesicular respiration replacing to a greater or less degree morbid breath-sounds. In those cases which were classed as cured, the departure from health being only such as is due to the results of every continued pneumonic process.

3. Secretion was diminished even in the cases marked only improved, and entirely absent in others.

4. Bacteriological reports in most cases bore out the results obtained in physical and other examinations.

5. The general condition of the patients improved in the large majority of cases, even in those whose physical examination did not show any great improvement.

6. The use of the remedy was not attended with any danger to the patient.

7. Finally, Antiphthisin does seem to have *curative* and not simply palliative qualities.

